# Association between *TNF-α* (−308G > A) promoter polymorphism and HHV-6 DNA detection in a community-based Thai cohort

**DOI:** 10.3389/fmicb.2026.1825548

**Published:** 2026-06-17

**Authors:** Sureewan Bumrungthai, Sutida Pongpukdeesakul, Tipaya Ekalaksananan, Sureewan Duangjit, Chamsai Pientong, Nuchsupha Sunthamala

**Affiliations:** 1 Division of Biopharmacy, Faculty of Pharmaceutical Sciences, Ubon Ratchathani University, Ubon Ratchathani, Thailand; 2 Division of Microbiology and Parasitology, School of Medical Sciences, University of Phayao, Phayao, Thailand; 3 Department of Microbiology, Faculty of Medicine, Khon Kaen University, Khon Kaen, Thailand; 4 Division of Pharmaceutical Chemistry and Technology, Faculty of Pharmaceutical Sciences, Ubon Ratchathani University, Ubon Ratchathani, Thailand; 5 Department of Biology, Faculty of Science, Mahasarakham University, Mahasarakham, Thailand; 6 Epidemic Simulation and Aetiology Nexus for Infectious Diseases, Mahasarakham University, Mahasarakham, Thailand

**Keywords:** cytokine gene polymorphism, human herpesvirus 6 (HHV-6), molecular epidemiology, TNF polymorphisms, viral latency and reactivation

## Abstract

**Background:**

Human herpesvirus 6 (HHV-6) establishes lifelong latency after primary infection and may reactivate under conditions of immune modulation. Tumor necrosis factor alpha (TNF-*α*) is a key pro-inflammatory cytokine involved in antiviral responses, and functional variation in the *TNF-α* promoter (−308G > A, rs1800629) may influence host–virus equilibrium. However, population-based data examining host genetic determinants of HHV-6 detection in Southeast Asia remain limited.

**Methods:**

We conducted a community-based cross-sectional study of 852 participants aged 3–90 years in Phayao Province, Thailand. HHV-6 DNA was detected by nested PCR and quantitative PCR. Genotyping of *TNF-α* (rs1800629) and additional polymorphisms was performed using high-resolution melt analysis with sequencing validation. Sociodemographic, clinical, and psychological variables were assessed using standardized questionnaires. Multivariable logistic regression was used to evaluate independent predictors of HHV-6 positivity.

**Results:**

HHV-6 DNA was detected in 12.8% of participants and increased with age (adjusted OR per 10-year increase 1.55, 95% CI 1.35–1.78; *p* < 0.001). The *TNF-α* (−308G > A) polymorphism was independently associated with HHV-6 detection (adjusted OR 3.21, 95% CI 1.98–5.20; *p* < 0.001). Lower education level also remained significant (adjusted OR 3.88, 95% CI 1.35–11.15; *p* = 0.012). The *DAT1* (rs40184) genotype showed a modest association in the full model (adjusted OR 1.84, 95% CI 1.02–3.32; *p* = 0.041) but was borderline in adult-only sensitivity analysis. Psychological measures were not independently associated with HHV-6 positivity.

**Conclusion:**

In this Thai community cohort, the *TNF-α* (−308G > A) promoter variant was independently associated with HHV-6 DNA detection. These findings support an observational model in which host immunogenetic variation contributes to inter-individual differences in viral DNA detection, although longitudinal studies are required to clarify causal mechanisms.

## 1 Introduction

Human herpesvirus 6 (HHV-6) is a ubiquitous beta-herpesvirus that establishes lifelong latency following primary infection, which is typically acquired in early childhood. After primary infection, HHV-6 persists in host cells, including monocytes, salivary gland tissue, and possibly the central nervous system, with the capacity for periodic reactivation under conditions of immune modulation (Padgett et al., 1998; Oakley and Cidlowski, 2013; Noisakran et al., 1998; Dhabhar, 2014; Dhabhar et al., 2012; Cohen et al., 1999; Tamai et al., 2022). Reactivation of latent herpesviruses is influenced by host immune status, inflammatory signaling, and glucocorticoid pathways, which collectively regulate viral gene expression and replication. Experimental evidence indicates that herpesvirus reactivation can be triggered by physiological stressors and immune suppression, although population-level determinants of HHV-6 DNA detection remain incompletely defined (Padgett et al., 1998; Oakley and Cidlowski, 2013; Noisakran et al., 1998; Dhabhar, 2014; Cain and Cidlowski, 2017; Jones, 2023). Environmental surveillance in northern Thailand has identified circulating HHV-6 variants in community water sources, suggesting ongoing exposure within certain rural settings (Pongpakdeesakul et al., 2023).

Studies in Asia have demonstrated widespread HHV-6 exposure across populations. Early seroepidemiological studies reported high infection rates during childhood, with nearly universal primary infection by age two (Yamanishi et al., 1990). Subsequent molecular studies identified HHV-6 DNA in peripheral blood among adults in several Asian populations, including China (Zhang et al., 2003) and Korea (Choi et al., 2005), reflecting viral persistence and latent infection dynamics. However, contemporary population-based data on HHV-6 DNA detection in blood samples from Thai cohorts remain limited. Recent work in Thailand has instead focused on buccal mucosa samples, where HHV-6 DNA was detected across different population groups, including patients with major depressive disorder and controls (Sumala et al., 2023). While these findings confirm the presence of HHV-6 in mucosal compartments, evidence from systemic (blood-based) sampling remains scarce. This gap underscores the need for updated studies using blood-derived samples to better characterize HHV-6 epidemiology in Thailand.

Tumor necrosis factor alpha (TNF-*α*) is a central pro-inflammatory cytokine involved in antiviral defense, immune activation, and regulation of NF-κB signaling pathways. TNF-α may influence herpesvirus biology directly or indirectly through inflammatory cascades and cellular stress responses (Rhen and Cidlowski, 2005; Busillo and Cidlowski, 2013; Vasileios et al., 2019; Yang et al., 2015). The functional promoter polymorphism −308G > A (rs1800629) has been associated with altered transcriptional activity and inter-individual variability in cytokine production. Variability at this locus may therefore influence host–virus equilibrium during latent infection and contribute to differences in viral detectability. Although TNF-*α* has been widely studied in inflammatory and infectious conditions, its relationship with HHV-6 DNA detection in general population settings has not been systematically evaluated.

Population-level data examining host genetic variation in relation to HHV-6 DNA detection remain limited, particularly in Southeast Asia. Most HHV-6 studies have focused on immunocompromised individuals or specific clinical populations, with relatively few investigations conducted in general community cohorts. In Thailand, epidemiological evidence of HHV-6 circulation exists (Pongpakdeesakul et al., 2023), yet the contribution of host cytokine gene polymorphisms to viral detection patterns remains poorly characterized. Understanding host genetic determinants of HHV-6 detection in endemic settings may provide important insight into viral persistence dynamics and immune regulation at the population level.

Therefore, the present study aimed to investigate the association between host genetic polymorphisms and HHV-6 DNA detection in a community-based cohort from Phayao Province, northern Thailand. Specifically, we examined the *TNF-α* (−308G > A) polymorphism and selected additional variants using targeted genotyping approaches and assessed their relationship with HHV-6 DNA detection by nested PCR and quantitative PCR. Multivariable logistic regression analyses were performed to determine whether *TNF-α* genotype was independently associated with HHV-6 positivity after adjustment for demographic and clinical covariates.

## 2 Materials and methods

### 2.1 Study population, ethical approval, and peripheral blood collection

Human peripheral blood samples (*n* = 852) were collected from individuals residing in Phayao Province, northern Thailand, between 2022 and 2023. Sample size estimation was based on a previously published study (Pongpakdeesakul et al., 2023). Participants were aged 3–90 years and self-identified as Thai ethnicity.

Demographic data and clinical characteristics were obtained using standardized questionnaires. Psychological stress was assessed using the Srithanya Stress Test (ST-5), a validated five-item instrument with scores ranging from 0 to 15 (Chomchoei et al., 2020). Depressive symptoms were screened using the Depression Questionnaire (Q2) (Paramee, 2026) and the Patient Health Questionnaire-9 (PHQ-9) (Paramee, 2026; Bains and Abdijadid, 2024; Malhi and Mann, 2018). These instruments were used for descriptive and covariate analyses.

Individuals with a clinical diagnosis of major depressive disorder (MDD) at the time of enrollment were excluded from the present analysis. Peripheral blood specimens were collected under aseptic conditions from community-based participants through local outreach activities in Mae Ka and Wiang subdistricts of Phayao Province. Approximately 3 mL of peripheral blood was drawn into EDTA tubes by a licensed medical technologist under aseptic conditions. Samples were transported to the laboratory in a temperature-controlled cold box to preserve sample integrity prior to processing.

This study was approved by the University of Phayao Human Research Ethics Committee (UP-HEC 1.3/023/63 and 1.3/013/65).

### 2.2 DNA extraction

Genomic DNA was extracted from 300 μL of whole blood using a commercial spin-column–based kit (PDC11-0100; BIO-HELIX, Taiwan) according to the manufacturer’s instructions (Pongpakdeesakul et al., 2023). The protocol included chemical lysis and heat incubation steps to ensure effective disruption of host cells and release of viral DNA. Purification was performed using silica-based columns, followed by washing and elution steps. Extracted DNA was stored at −20 °C until further analysis.

### 2.3 HHV-6 DNA detection by nested PCR and quantitative PCR

HHV-6 DNA was detected by nested PCR targeting the U97 gene, as previously described (Sumala et al., 2023). The first-round PCR amplified a 520 bp fragment, and the second-round PCR amplified a 259 bp fragment. PCR products were visualized on 2% agarose gels.

Quantitative PCR (qPCR) was performed for confirmation and viral load determination using standard protocols (Sumala et al., 2023). An in-house plasmid containing the HHV-6 U97 gene (GenBank accession AB021506.1) was used as a positive control and for standard curve generation. Viral copy numbers were calculated using established formulas based on plasmid concentration and amplicon length (Sumala et al., 2023).

The human *beta-globin* gene was used as an internal amplification control (IAC) to confirm DNA integrity and monitor potential PCR inhibition (Bon et al., 2000). Samples failing *beta-globin* amplification were excluded from HHV-6 analyses. All reactions were performed in duplicate to triplicate.

To minimize the risk of contamination, pre- and post-amplification steps were conducted in physically separated areas with dedicated equipment and consumables. DNA extraction, PCR setup, and amplification were performed in designated workspaces. In addition, negative and positive controls were included in each batch of nested PCR and qPCR assays, as well as during DNA extraction, to monitor assay performance and detect potential contamination.

### 2.4 SNP genotyping by high-resolution melt (HRM) analysis

Single nucleotide polymorphisms (SNPs) in *TNF-α* (−308G > A, rs1800629), *DAT1* (rs40184), *JARID2* (rs9383046), and *SLC6A3* (rs6345) were genotyped using high-resolution melt (HRM) analysis. Primer sequences are shown in Table 1.

**Table 1:** Primers for PCR and real time PCR (HRM).

Primer	Sequence	Primers size	Reference
*HHV-6 outer primer*	5’-GCGTTTTCAGTGTGTAGTTCGGCAG-3′	520 bp	Sumala et al. (2023)
	5’-TGGCCGCATTCGTACAGATACGGAGG-3′		
*HHV-6 inner primer*	5’-GCTAGAACGTATTTGCTGCAGAACG-3’	258 bp	Sumala et al. (2023)
	5’-ATCCGAAACAACTGTCTGACTGGCA-3’		
*B-globin*	GAAGAGCCAAGGACAGGTAC 3′	268 bp	Bon et al. (2000)
	5′ CAACTTCATCCACGTTCACC 3′		
*TNF-α rs1800629*	5*′*CACAGACCTGGTCCCCAAAA 3*′*	136 bp	Sumala et al. (2023)
	5*′* CATCCTCCCTGCTCCGATTC 3*′*		
*DAT1* rs40184	5’-CACAGTCTCGCGGCTTTT-3′	100 bp	Faris et al. (2018)
	5’-TGGACCAACACACCCTTGA-3′		
*JARID2* rs9383046	5’-ACTGGCTGTGT-CTCACTCTT-3′	88 bp	Chen et al. (2017)
	5’-TATTCACGTTCTTTTG-CTCTTGGA-3′		
*JARID2* rs9383046*	5’-ATTTGACCCACACTGGCTGT-3′	96 bp	Bumrungthai et al. (2024)
	5’-TCACGTTCTTTTGCTCTTGGAC-3′		
*SLC6A3* rs6345	5’-GTAGAGCAGCACGATGACCA-3′	93 bp	Bumrungthai et al. (2024)
	5’-CTGCACCTCCACCAGAGC-3′		
*SLC6A3* rs6345*	5’-GCTGAAGTAGAGCAGCACGA-3′	166 bp	Bumrungthai et al. (2024)
	5’-CAGTTCCAGGTGGGTTGACA-3’		

*Outer primer for sequencing; *DAT1* and *TNF-α* used for HRM and sequencing.

HRM assays were conducted in duplicate following previously published protocols (Sumala et al., 2023). Genotypes were determined based on normalized melting curves and characteristic melting temperature (Tm) profiles. Distinct melting curve profiles were clustered using instrument software and further verified by manual inspection, with genotype clusters defined by reproducible curve shapes and Tm differences (typically 0.2–0.5 °C depending on SNP context) observed across samples. Positive control samples confirmed by sequencing were included in each run. All HRM profiles were independently reviewed, and ambiguous or borderline melting curves were reanalyzed to ensure consistent genotype classification and minimize potential genotyping error.

### 2.5 Sanger sequencing validation

To validate HRM-based genotyping results, a subset of approximately 20–30 samples representing each genotype cluster was randomly selected for Sanger sequencing. PCR amplification was performed using the primers described in Section 2.4. The resulting PCR products were sequenced and aligned with reference sequences from GenBank (GRCh38.p13; NC_000006.12) using BioEdit version 7.2 for genotype confirmation. The sequencing results were fully concordant with HRM-based genotype assignments, supporting the accuracy of the HRM genotyping approach.

### 2.6 Serum TNF-*α* measurement

Serum TNF-α levels were measured using a commercial human TNF-α ELISA kit (BD Biosciences, USA) according to the manufacturer’s instructions. Samples were analyzed in duplicate under blinded conditions. Absorbance was measured at 450 nm, and concentrations were calculated from standard curves.

### 2.7 Exploratory serum proteomic analysis

Exploratory serum proteomic profiling was performed using liquid chromatography–mass spectrometry (LC–MS) as previously described (Pongpakdeesakul et al., 2023). Serum samples were pooled according to stress status, HHV-6 detection, and *TNF-α* genotype. Proteins were reduced, alkylated, digested with trypsin, and analyzed using an Agilent 6545XT QTOF system. Protein identification was conducted using peptide-spectrum matching with a 1% false discovery rate (FDR) threshold at the protein level. Due to the pooled design, proteomic results were considered descriptive and hypothesis-generating.

### 2.8 Statistical analysis

Statistical analyses were performed using IBM SPSS version 16. Categorical variables were compared using Pearson’s chi-squared test, and continuous variables were assessed for normality using the Shapiro–Wilk test. Viral load distributions were right-skewed and therefore log10-transformed prior to parametric comparison; nonparametric tests were used when assumptions were not met. TNF concentrations were analyzed using Mann–Whitney U test due to non-normal distribution. Hardy–Weinberg equilibrium was assessed using the chi-square test (or exact test when appropriate). Logistic regression analyses were performed to assess associations between genetic polymorphisms and HHV-6 positivity. Candidate covariates included age, sex, stress score, depression score, BMI, and education level based on prior biological plausibility and univariate screening (*p* < 0.20). Adjusted odds ratios (ORs) and 95% confidence intervals (CIs) were calculated. A *p*-value < 0.05 was considered statistically significant. Psychological variables were evaluated as candidate confounders and retained only if they were independently associated with HHV-6 positivity or meaningfully changed the main effect estimates. Genotype-specific sample sizes varied due to missing DNA or incomplete questionnaire data; analyses were performed using available-case data for each variable.

## 3 Results

### 3.1 Participant characteristics

A total of 852 participants were included in the primary analysis. The cohort consisted of 606 females (71.1%) and 246 males (28.9%), with ages ranging from 3 to 90 years. The largest age groups were 51–60 years (*n* = 202) and 61–70 years (*n* = 219). Overall, HHV-6 DNA was detected in 109 participants (12.8%). HHV-6 positivity increased markedly with age (*p* < 0.001). No positive cases were detected among individuals aged ≤20 years. The highest positivity rate was observed in participants aged 51–60 years (29.7%) (Table 2).

HHV-6 detection was not significantly associated with stress or depression based on questionnaire data. However, HHV-6 positivity was significantly associated with *DAT1* (C > T) and *TNF-α* (G > A) polymorphisms, suggesting an increased risk of HHV-6 DNA detection among individuals carrying these variants (Table 2).

**Table 2:** Factors Associated with HHV-6 DNA Detection (Univariate Analysis).

**Factor**	**Category**	***N***	**HHV-6 positive *n* (%)**	**HHV-6 negative *n* (%)**	**OR (95% CI)**	***p*-value**
Sex	Male	246	38 (15.5)	208 (84.5)	1.38 (0.90–2.11)	0.140
	Female (ref)	606	71 (11.7)	535 (88.3)	—	—
Age group (years)	3–10	21	0 (0)	21 (100)	NE	<0.001*
	11–20	63	0 (0)	63 (100)	NE	
	21–30 (ref)	76	1 (1.3)	75 (98.7)	1.00 (−)	
	31–40	54	3 (5.6)	51 (94.4)	4.41 (0.45–43.2)	
	41–50	121	13 (10.7)	108 (89.3)	9.07 (1.16–70.9)	
	51–60	202	60 (29.7)	142 (70.3)	54.3 (7.4–397)	
	61–70	219	27 (12.3)	192 (87.7)	11.2 (1.47–85.7)	
	71–80	76	3 (4.0)	73 (96.0)	3.09 (0.31–30.7)	
	81–90	20	2 (10.0)	18 (90.0)	8.33 (0.73–94.8)	
Education level	≤ High vocational certificate	735	105 (14.3)	632 (85.7)	4.61 (1.67–12.77)	0.001
	> High vocational certificate (ref)	115	4 (3.5)	111 (96.5)	—	—
Income/month (USD)	300–1,000	763	101 (13.2)	662 (86.8)	1.55 (0.73–3.29)	0.256
	≤299 (ref)	89	8 (9.0)	81 (91.0)	—	—
BMI	16–25	662	92 (13.9)	570 (86.1)	1.85 (1.02–3.32)	0.039
	<16 or >25 (ref)	174	14 (8.1)	160 (91.9)	—	—
Congenital disease	Yes	474	67 (14.1)	407 (85.9)	1.32 (0.87–1.99)	0.189
	No (ref)	378	42 (11.1)	336 (88.9)	—	—
Exercise	3–7 times/week	419	60 (14.3)	359 (85.7)	1.31 (0.88–1.95)	0.819
	≤1–2 times/week (ref)	433	49 (11.3)	384 (88.7)	—	—
Alcohol consumption	Yes	309	47 (15.2)	262 (84.8)	1.39 (0.92–2.09)	0.113
	No (ref)	542	62 (11.4)	480 (88.6)	—	—
Smoking	Yes	144	20 (13.9)	124 (86.1)	1.12 (0.67–1.89)	0.670
	No (ref)	707	89 (12.6)	618 (87.4)	—	—
ST-5 stress	≥5	168	19 (11.3)	149 (88.7)	—	0.827
	0–4 (ref)	679	89 (13.1)	590 (86.9)	—	—
Q2	Yes	178	24 (13.5)	154 (86.5)	1.10 (0.68–1.80)	0.696
	No (ref)	654	81 (12.4)	573 (87.6)	—	—
PHQ-9	≥7	64	11 (17.2)	53 (82.8)	—	0.436
	0–6 (ref)	788	98 (12.4)	690 (87.6)	—	—
*DAT1* (rs40184)	C	594	93 (15.7)	501 (84.3)	2.22 (1.25–3.92)	0.005
	C > T (ref)	194	15 (7.7)	179 (92.3)	—	—
*TNF-α* (rs1800629)	G > A	121	36 (29.8)	85 (70.2)	3.69 (2.33–5.85)	<0.001
	G (ref)	700	72 (10.3)	628 (89.7)	—	—
*JARID2* (rs9383046)	G > A	2	0 (0)	2 (100)	—	0.586
	G (ref)	842	109 (13.0)	733 (87.0)	—	—
*SLC6A3* (*DAT1*) (rs6345)	C (ref)	749	96 (12.8)	653 (87.2)	—	—

* Age compared across categories by chi-square trend test. OR not estimable due to zero events. Not estimable (NE).

### 3.2 Metagenomic identification of HHV-6A

**Figure 1:**
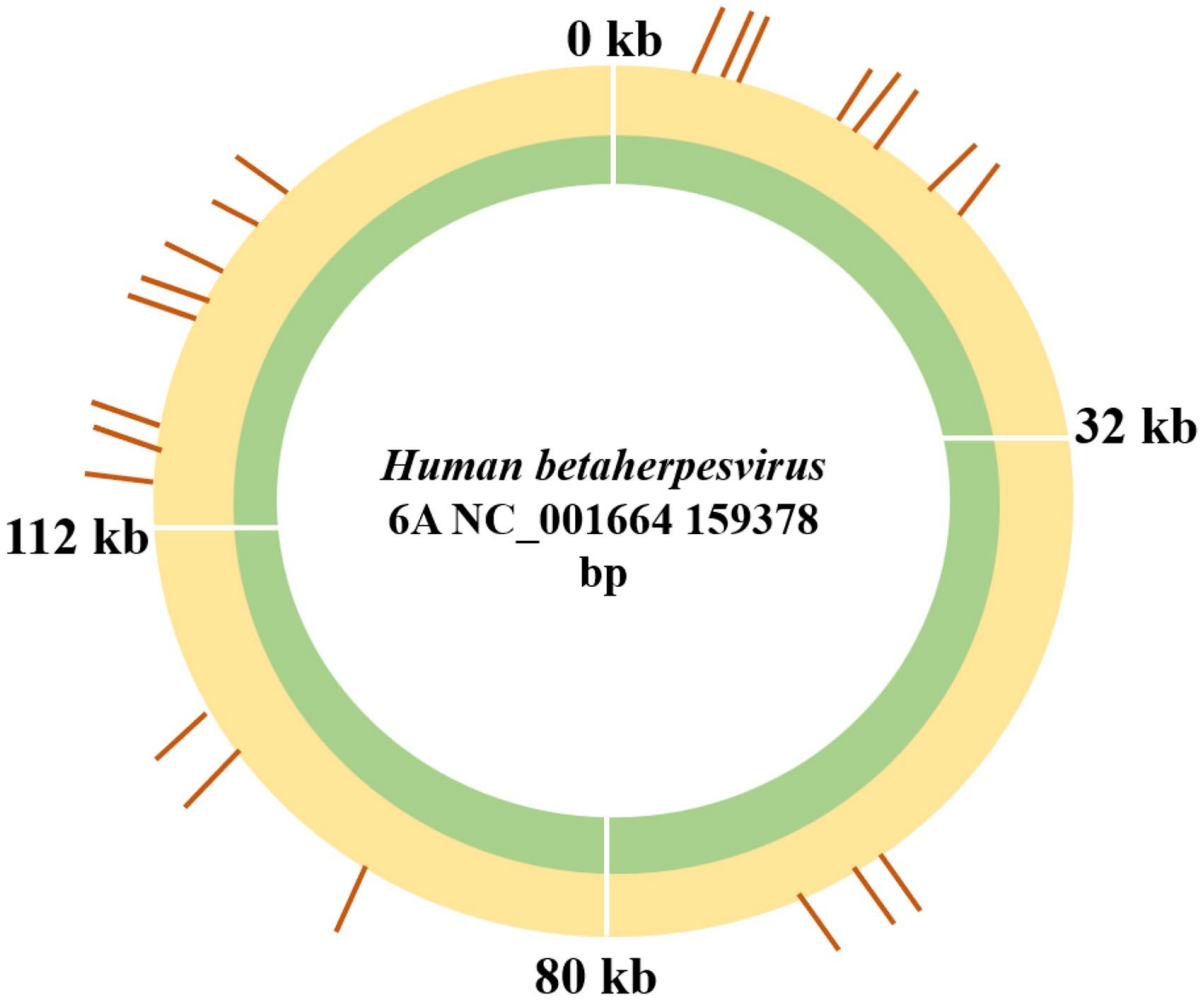
Metagenomic mapping of HHV-6 sequences detected in whole-blood samples. Circular genome map showing metagenomic sequence reads mapped to the reference genome of Human betaherpesvirus 6A (HHV-6A; GenBank accession NC_001664; 159,378 bp). The concentric rings represent the viral genome coordinates, with annotated positions indicated in kilobases (kb). Brown radial lines denote metagenomic sequence fragments identified from pooled whole-blood samples and aligned to corresponding locations within the HHV-6 genome. This visualization illustrates the genomic distribution of detected HHV-6 sequences without implying viral replication activity, temporal dynamics, or functional consequences.

HHV-6 DNA was initially evaluated using shotgun metagenomic sequencing data derived from pooled whole-blood samples (*n* = 852) from the normal Phayao population. Metagenomic analysis identified sequences corresponding to *Human betaherpesvirus* 6A, including regions *DR1, U2, U3, U4, U7, U10, U11, U12,* and *U13* (positions 0–18,000 bp), as well as *U79, U81, U82, U83A, U84, U85, U86, R1, U91, U94, U95, U100A, U100B,* and *DR* (positions 112,000–144,000 bp) (Figure 1 and Supplementary Table S1). Metagenomic mapping was performed on pooled samples and therefore does not provide individual-level viral quantification.

### 3.3 Sociodemographic and clinical factors associated with HHV-6 detection

Univariate analyses were conducted to evaluate associations between participant characteristics and HHV-6 DNA detection. HHV-6 positivity was higher in males (15.45%) than in females (11.72%); however, this difference did not reach statistical significance (OR = 1.38, 95% CI 0.90–2.11, *p* = 0.140). Education level was significantly associated with HHV-6 detection. Participants with education up to high vocational certificate level demonstrated a higher prevalence of HHV-6 positivity (14.29%) compared with those with education beyond this level (3.48%) (OR = 4.61, 95% CI 1.67–12.77, *p* = 0.001). Body mass index (BMI) was also associated with HHV-6 detection. Individuals with BMI between 16 and 25 had a higher prevalence of HHV-6 positivity (13.90%) compared with those outside this range (8.05%) (OR = 1.85, 95% CI 1.02–3.32, *p* = 0.039). No significant associations were observed for income level, familial relationship status, congenital disease, exercise frequency, alcohol consumption, smoking status, ST-5 stress score, Q2 score, or PHQ-9 category in univariate analyses (Table 2).

### 3.4 Distribution of psychological measures in the study population

Psychological stress and depressive symptoms were assessed using the ST-5, PHQ-9, and Q2 instruments for descriptive and covariate purposes. Based on ST-5 scores, 80% of participants reported no stress, while 20% demonstrated elevated stress levels, including mild-to-moderate (16%), moderate (2%), and high stress (2%) categories (Supplementary Figure S1A). According to PHQ-9 classification, 93% of participants had no depressive symptoms, whereas 6 and 1% were categorized as having mild and moderate depressive symptoms, respectively; no cases met criteria for major depression (Supplementary Figure S1B). Q2 screening indicated that 21% of participants were classified as being at risk of depression, while 79% reported no depressive symptoms (Supplementary Figure S1C). For regression modeling, stress and depression variables were collapsed into binary categories to improve statistical stability.

### 3.5 Genetic polymorphism distribution and association with HHV-6 detection

Genotype distributions of selected polymorphisms were evaluated in the cohort. The TNF-*α* − 308G > A (rs1800629) polymorphism was observed in 121 of 821 individuals (14.7%), while the wild-type genotype was present in 700 individuals (85.3%). The *DAT1* (rs40184) variant was detected in 194 of 788 individuals (24.6%). The *JARID2* (rs9383046) and *SLC6A3* (rs6345) variants were rare or absent in this population (Table 2). Genotype distributions for *TNF-α* and *DAT1* polymorphisms deviated from Hardy–Weinberg equilibrium (*p* < 0.05), and detailed HWE test results are provided in Supplementary Table S4.

None of the examined polymorphisms showed significant associations with ST-5 stress levels, Q2 screening status, or PHQ-9 depression categories (Supplementary Table S2). In contrast, HHV-6 positivity was significantly more frequent among individuals carrying the *TNF-α* G > A genotype (36/121, 29.8%) compared with those carrying the wild-type *TNF-α* genotype (72/700, 10.3%) (*p* < 0.001). Univariate logistic regression demonstrated a strong association between *TNF-α* G > A and HHV-6 detection (OR = 3.69, 95% CI 2.33–5.85).

The *DAT1* genotype, BMI, and education level were also associated with HHV-6 detection in univariate analysis (Table 2). However, in univariate logistic regression models adjusted for age, sex, BMI, and education level, the *TNF-α* G > A polymorphism remained independently associated with HHV-6 positivity (adjusted OR = 3.69, 95% CI 2.33–5.85, *p* < 0.001) (Table 2). Markedly higher odds were observed in middle-aged groups; however, category-specific ORs should be interpreted cautiously due to sparse reference cells. Because HHV-6 positivity was absent in individuals ≤20 years and rare in 21–30 years, age category–specific odds ratios should be interpreted cautiously due to sparse cell counts. These findings demonstrate that the *TNF-α* (−308G > A) polymorphism is independently associated with HHV-6 detection in this community-based cohort.

### 3.6 Multivariable logistic regression analysis

To determine independent predictors of HHV-6 positivity, a multivariable logistic regression model was constructed including age (continuous), sex, education level, BMI category, *TNF-α* (rs1800629) genotype, and *DAT1* (rs40184) genotype. Psychological variables (ST-5, Q2, and PHQ-9) were initially included as potential confounders; however, they were not independently associated with HHV-6 positivity and did not materially change the effect estimates of the primary predictors. These variables were therefore excluded from the final parsimonious model. After adjustment for all covariates, the *TNF-α* (−308G > A) polymorphism remained strongly and independently associated with HHV-6 detection. Individuals carrying the G > A genotype had more than a threefold increased odds of HHV-6 positivity compared with those carrying the wild-type G genotype (adjusted OR = 3.21, 95% CI 1.98–5.20, *p* < 0.001) (Table 3). Education level also remained independently associated with HHV-6 detection. Participants with education up to high vocational certificate level had significantly higher odds of HHV-6 positivity compared with those with higher education (adjusted OR = 3.88, 95% CI 1.35–11.15, *p* = 0.012) (Table 3). The *DAT1* (rs40184) genotype showed a modest but statistically significant association in the adjusted model. Individuals carrying the C genotype had increased odds of HHV-6 positivity compared with C > T carriers (adjusted OR = 1.84, 95% CI 1.02–3.32, *p* = 0.041) (Table 3). BMI category was not independently associated with HHV-6 detection after adjustment (adjusted OR = 1.62, 95% CI 0.88–2.98, *p* = 0.118). Sex was also not significantly associated with HHV-6 positivity (adjusted OR = 1.25, 95% CI 0.80–1.95, *p* = 0.320) (Table 3). In the full cohort, age was independently associated with HHV-6 detection. Each 10-year increase in age was associated with approximately 55% higher odds of HHV-6 positivity (adjusted OR 1.55, 95% CI 1.35–1.78, *p* < 0.001) (Table 3). Overall, these findings indicate that the *TNF-α* (−308G > A) polymorphism is an independent host genetic factor associated with HHV-6 detection in this community-based cohort, even after accounting for demographic and socioeconomic variables.

**Table 3:** Multivariable logistic regression analysis of factors associated with HHV-6 positivity (*N* = 852).

Variable	Comparison	Adjusted OR	95% CI	*p*-value
Age	Per 10-year increase	1.55	1.35–1.78	<0.001
Sex	Male vs. Female (ref)	1.25	0.80–1.95	0.320
Education level	≤High vocational vs. >High vocational (ref)	3.88	1.35–11.15	0.012
BMI	16–25 vs. <16 or >25 (ref)	1.62	0.88–2.98	0.118
*TNF-α* (rs1800629)	G > A vs. G (ref)	3.21	1.98–5.20	<0.001
*DAT1* (rs40184)	C vs. C > T (ref)	1.84	1.02–3.32	0.041

Outcome: HHV-6 DNA detection (1 = positive, 0 = negative). * Only samples with complete covariate and genotype data were included in the final model. Odds ratios were adjusted simultaneously for all variables listed in the table. Age was entered as a continuous variable. Reference categories: Female sex, > high vocational education, BMI <16 or >25, *TNF-α* (G) genotype, and *DAT1* (C > T) genotype. Statistical significance was defined as *p* < 0.05.

### 3.7 Age-dependent HHV-6 prevalence and distribution of viral load and TNF-α levels

**Figure 2:**
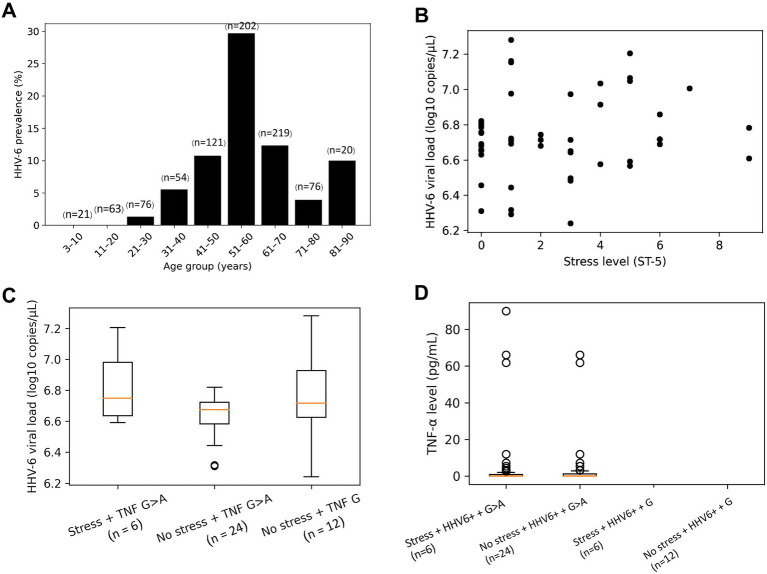
Age-dependent prevalence and distribution of HHV-6 viral load and TNF-α levels in relation to stress and *TNF-α* genotype. **(A)** Prevalence of HHV-6 DNA detection across age groups in the community-based cohort (*N* = 852). Bars represent the percentage of HHV-6–positive individuals within each age category. Sample sizes for each age group are indicated above the bars. **(B)** Distribution of HHV-6 viral load among positive individuals according to stress level assessed by the ST-5 questionnaire. Viral load is expressed as log10 copies/μL. Each point represents an individual sample (*n* = 52). **(C)** HHV-6 viral load (log10 copies/μL) stratified by stress status and *TNF-α* (−308G > A) genotype. Boxes represent median and interquartile range (IQR), whiskers indicate data dispersion, and points represent outliers. Sample sizes for each subgroup are indicated in the figure. **(D)** Circulating *TNF-α* concentrations (pg/mL) according to combined stress status and *TNF-α* genotype among HHV-6–positive individuals. Boxes represent median and IQR, whiskers indicate data dispersion, and individual points represent outliers. Sample sizes for each subgroup are indicated in the figure. Data are presented descriptively to illustrate distributional differences and do not imply causality or temporal relationships.

HHV-6 DNA prevalence increased significantly with age in the community-based cohort (Figure 2A). No positive cases were detected among participants aged ≤20 years, whereas prevalence increased progressively in older age groups, reaching a peak of 29.7% in individuals aged 51–60 years. A significant age-dependent trend was observed (*p* < 0.001), consistent with cumulative exposure or age-related immune modulation. Among the 109 HHV-6–positive individuals, viral load was quantifiable in 52 cases (47.7% of HHV-6–positive individuals). Viral load values, expressed as log10 copies/μL, demonstrated substantial inter-individual variability (Figure 2B and Supplementary Table S2). No significant association was observed between stress level (ST-5 score) and HHV-6 viral load (*p* = 0.147), and no clear linear or monotonic trend was evident.

When stratified by stress status and *TNF-α* (−308G > A) genotype, viral load distributions showed modest differences (Figure 2C). HHV-6–positive individuals with stress exhibited numerically higher viral loads compared with non-stressed individuals; however, substantial overlap was observed between groups. Subgroup analysis indicated that HHV-6–positive individuals with stress carrying either *TNF-α* genotype had higher viral loads than non-stressed HHV-6–positive individuals with the wild-type *TNF-α* genotype (*p* = 0.024). Given the limited number of quantifiable cases and the cross-sectional design, these findings should be interpreted descriptively. Circulating TNF-α concentrations were further examined across stress status, HHV-6 detection, and *TNF-α* genotype (Figure 2D and Supplementary Table S2). Individuals without stress who carried the *TNF-α* (G > A) polymorphism exhibited higher TNF-α levels than those with the wild-type genotype (*p* = 0.022). Overall, TNF-α levels were significantly lower in HHV-6–positive individuals compared with HHV-6–negative individuals (*p* = 0.015). Among individuals without stress carrying the *TNF-α* (G > A) genotype, TNF-α concentrations were significantly lower in HHV-6–positive cases compared with HHV-6–negative cases (*p* = 0.007). No significant association was observed between TNF-*α* concentration and viral load magnitude. Collectively, Figure 2 illustrates an age-associated increase in HHV-6 prevalence, limited evidence for a direct association between stress and viral load, and genotype-associated differences in circulating TNF-α levels. These observations are descriptive and do not imply causal or directional relationships.

### 3.8 Sensitivity analysis restricted to adults (≥21 years)

To evaluate whether inclusion of pediatric participants influenced the primary findings, multivariable logistic regression analyses were restricted to adults aged ≥21 years. The adult subset comprised 759 individuals, of whom 741 were retained in the complete-case regression model; 104 participants were HHV-6 positive. In this adult-only model, the *TNF-α* (−308G > A) polymorphism remained independently associated with HHV-6 detection. Individuals carrying the G > A genotype had significantly higher odds of HHV-6 positivity compared with those carrying the wild-type G genotype (adjusted OR = 2.72, 95% CI 1.63–4.54, *p* < 0.001). For *DAT1* (rs40184), individuals carrying the C genotype demonstrated higher odds of HHV-6 positivity compared with C > T carriers; however, this association was of borderline statistical significance (adjusted OR = 1.67, 95% CI 0.97–2.86, *p* = 0.064). Age, entered as a continuous variable, was not independently associated with HHV-6 detection within the adult subset (adjusted OR per 1-year increase = 1.01, 95% CI 0.99–1.02, *p* = 0.471). Male sex (vs female), ST-5 stress score, Q2 score, and PHQ-9 score were also not significantly associated with HHV-6 positivity in the adjusted model (Table 4). These findings indicate that the association between the *TNF-α* (−308G > A) polymorphism and HHV-6 detection is robust and not driven by the inclusion of younger participants. The age association observed in the full cohort appears to reflect the structural absence of HHV-6 positivity among individuals ≤20 years rather than a continuous increase in risk across adulthood. The absence of detectable HHV-6 DNA among younger participants may reflect either reduced circulating viral DNA following primary infection or methodological sensitivity limitations in low-viremia states, rather than complete absence of latent infection.

**Table 4:** Sensitivity analysis: multivariable logistic regression restricted to adults (≥21 years).

**Variable**	**Comparison**	**Adjusted OR**	**95% CI**	**p-value**
Sex	Male vs. Female (ref)	1.27	0.76–2.13	0.364
Age (years)	Per 1-year increase	1.01	0.99–1.02	0.471
ST-5 score	Per 1-point increase	1.01	0.86–1.19	0.892
Q2 score	Per 1-point increase	0.86	0.64–1.16	0.319
PHQ-9 score	Per 1-point increase	1.04	0.99–1.09	0.117
*TNF-α* (rs1800629)	G > A vs. G (ref)	2.72	1.63–4.54	<0.001
*DAT1* (rs40184)	C vs. C > T (ref)	1.67	0.97–2.86	0.064

Outcome: HHV-6 DNA detection (1 = positive, 0 = negative). Adult sample: *n* = 759. Complete-case model: *n* = 741 (HHV-6 positive = 104). Odds ratios were adjusted simultaneously for all variables listed in the table. Age, ST-5, Q2, and PHQ-9 were entered as continuous variables. Reference categories: Female sex, *TNF-α* (G) genotype, and *DAT1* (C > T) genotype. Only participants with complete covariate and genotype data were included. Statistical significance was defined as *p* < 0.05.

### 3.9 Hardy–Weinberg equilibrium analysis

Hardy–Weinberg equilibrium (HWE) was assessed for each polymorphism using exact tests based on observed genotype frequencies in the full cohort, as this study was conducted in a population-based sample rather than a case–control design. For *TNF-α* (rs1800629), genotype distribution included 700 major homozygotes (G/G), 121 heterozygotes (G/A), and no minor homozygotes (A/A) among 821 successfully genotyped individuals. This distribution deviated from HWE expectations (exact *p* = 2.29 × 10^−4^). Similarly, *DAT1* (rs40184) genotype counts comprised 594 major homozygotes (C/C), 194 heterozygotes (C/T), and no minor homozygotes (T/T) among 788 individuals, also demonstrating deviation from HWE (exact *p* = 1.23 × 10^−8^). In contrast, *JARID2* (rs9383046) genotype frequencies (842 major homozygotes, 2 heterozygotes, 0 minor homozygotes; n = 844) were consistent with HWE (exact *p* = 0.998). For both *TNF-α* and *DAT1*, deviation from equilibrium was primarily attributable to the absence of minor homozygous genotypes despite the presence of heterozygotes. Potential explanations include low minor allele frequency, sampling variation, population substructure, or non-random mating patterns within the study population. High-resolution melt (HRM) genotyping results were validated by Sanger sequencing in representative samples to minimize the likelihood of systematic genotyping error (Table 5).

**Table 5:** Hardy–Weinberg equilibrium (HWE) analysis of genotyped polymorphisms.

**SNP (rsID)**	**Major Homozygote (n)**	**Heterozygote (n)**	**Minor Homozygote (n)**	**Total (n)**	**Exact HWE p-value**
*TNF-α* (rs1800629)	700	121	0	821	2.29 × 10^−4^
*DAT1* (rs40184)	594	194	0	788	1.23 × 10^−8^
*JARID2* (rs9383046)	842	2	0	844	0.998

HWE was evaluated using exact tests based on observed genotype frequencies. Minor homozygous genotypes were not observed for *TNF-α* and *DAT1* in this cohort.

Detailed observed and expected genotype counts are provided in Supplementary Table S3 to facilitate transparent evaluation of equilibrium assumptions.

### 3.10 Exploratory proteomic profiling (supplementary analysis)

Exploratory pooled serum proteomic profiling was performed to assess differential protein abundance patterns according to stress status, HHV-6 detection, and *TNF-α* genotype. Given the pooled design, these analyses were descriptive and hypothesis-generating. Differential patterns involving immune-related and extracellular matrix–associated proteins were descriptively observed (Supplementary Table S4). No statistical inference was performed for proteomic comparisons.

## 4 Discussion

This community-based study in Phayao Province, northern Thailand, evaluated host and contextual factors associated with HHV-6 DNA detection and focused on the *TNF-α* − 308G > A (rs1800629) polymorphism as a candidate immunogenetic determinant. HHV-6 is a ubiquitous beta-herpesvirus that establishes lifelong latency after early-life infection, with reactivation and detectability influenced by immune and inflammatory signaling (Padgett et al., 1998; Oakley and Cidlowski, 2013; Noisakran et al., 1998; Dhabhar, 2014; Dhabhar et al., 2012; Cohen et al., 1999; Tamai et al., 2022). In this cohort, HHV-6 DNA was detected in 12.8% of participants, and detection varied strongly by age, with no positives among participants ≤20 years and higher positivity observed in mid-to-late adulthood, consistent with the concept that immune modulation and host–virus equilibrium may shift across the life course. The apparent age association in the full model reflects structural zero counts in younger strata rather than a continuous linear increase across adulthood.

Primary HHV-6 infection typically occurs in early childhood, with most individuals infected by the age of two. Therefore, the absence of HHV-6 DNA detection among participants ≤20 years in this study was unexpected. However, the number of individuals in this age group was relatively small (*n* = 84), which may have limited the statistical power to detect HHV-6 DNA. In addition, detection of HHV-6 DNA in blood depends on multiple factors, including viral load, specimen type, and DNA extraction efficiency. Viral DNA levels may have been below the analytical sensitivity of the qPCR assay, particularly in the absence of active viral replication (Collot et al., 2002). Importantly, HHV-6 DNA detection in blood reflects current detectability rather than prior exposure, and therefore the absence of detectable HHV-6 DNA should not be interpreted as absence of infection. Further studies with larger pediatric cohorts and optimized sampling strategies are warranted.

The observed HHV-6 detection frequency was comparable to reports from Vietnam (12.6%) (Tran et al., 2023) and within the lower range reported in China (16–40%) (Zheng et al., 2021), supporting the external plausibility of the detection rate in this setting. Beyond age, lower educational attainment (≤high vocational certificate) was associated with higher HHV-6 positivity in both univariate and multivariable models, suggesting that social or behavioral correlates linked to education may contribute to exposure patterns, immune health, or other unmeasured determinants of viral detectability. BMI showed an association in univariate analysis but was attenuated after adjustment, indicating that its effect may be confounded by other covariates or reflect limited discriminative value using the chosen BMI categories.

A key finding is that the *TNF-α* (−308G > A) polymorphism was independently associated with HHV-6 detection after adjustment for demographic and clinical covariates. TNF-*α* is a central pro-inflammatory cytokine that regulates antiviral defense and NF-κB–linked signaling pathways, which are relevant to herpesvirus latency and reactivation biology (Rhen and Cidlowski, 2005; Busillo and Cidlowski, 2013; Vasileios et al., 2019; Yang et al., 2015; Zhang and An, 2007). The *TNF-α* (−308G > A) promoter variant has been linked to altered transcriptional activity and inter-individual variability in TNF-α responses, offering a biologically plausible mechanism by which host genotype could influence HHV-6 persistence dynamics or the probability of detectable viremia at a given time point. While herpesvirus reactivation can be modulated by inflammatory and glucocorticoid pathways (Padgett et al., 1998; Oakley and Cidlowski, 2013; Noisakran et al., 1998; Dhabhar, 2014; Cain and Cidlowski, 2017; Jones, 2023), population-based evidence connecting *TNF-α* promoter variation to HHV-6 detection remains limited in Southeast Asia, and the present data help address this gap. TNF-α signaling exerts context-dependent effects in herpesvirus biology, influencing both antiviral responses and latency–reactivation dynamics. The *TNF-α* (−308G > A) promoter variant has been associated with altered transcriptional responsiveness and inter-individual variability in cytokine production. It is therefore plausible that genotype-specific differences in *TNF-α* regulation affect the probability of transient viral DNA detection in peripheral blood, rather than constitutive viral replication per se. This framework is consistent with an immunoregulatory threshold model in which host genetic variation influences equilibrium between immune containment and episodic viral expression.

While the detection of HHV-6 DNA in blood samples is meaningful for establishing viral presence, it cannot reliably distinguish between latent infection, chromosomal integration (ciHHV-6), transient viremia, or active viral replication. Chromosomally integrated HHV-6 (ciHHV-6), in which the entire viral genome is integrated into host chromosomes and present in all nucleated cells, occurs in approximately 1% of individuals and is associated with persistently high viral DNA loads in blood regardless of active infection status (Pellett et al., 2012; Ward et al., 2006). In addition, viral DNA detected by PCR may originate from lysed cells rather than active replication. Therefore, reliance on PCR-based detection in blood compartments alone is insufficient to define viral activity. Accordingly, the findings of this study should be interpreted as reflecting HHV-6 detectability rather than active infection, and mechanistic interpretations beyond presence of viral DNA should be avoided without complementary diagnostic or functional evidence.

We also evaluated additional polymorphisms previously reported in psychiatric-genetic contexts. The frequency of *DAT1* (rs40184) observed here was broadly consistent with other Asian populations (Yang et al., 2014). Although prior work (including our own) has reported differences in *DAT1* (rs40184) frequency between MDD cases and controls (Bumrungthai et al., 2024), we did not observe associations between the investigated polymorphisms and questionnaire-based stress or depression screening in this community cohort, which is consistent with the view that genetic susceptibility may manifest most clearly under sustained or repeated stress exposure or within clinical phenotypes rather than in low-severity population screening distributions (Ising and Holsboer, 2006). Importantly, psychological variables were evaluated as potential confounders in regression models, but they did not independently associate with HHV-6 positivity and did not materially alter the genetic effect estimates; therefore, they were not retained in the final parsimonious model.

Several limitations should be considered. First, the cross-sectional design supports association but not causality, and HHV-6 detection reflects a single sampling window rather than longitudinal reactivation patterns. Second, genotype distributions for *TNF-α* and *DAT1* deviated from Hardy–Weinberg equilibrium, largely driven by the absence of minor homozygotes despite observed heterozygotes; this may reflect low minor allele frequency, sampling variation, population structure, or limitations of genotype calling/categorization, even though HRM calls were supported by Sanger validation in representative samples. Third, HHV-6 subtype differentiation (A vs. B) was not incorporated, which could be relevant to epidemiology and immunobiology. Finally, unmeasured factors (e.g., comorbidities, medication use, or additional immune markers) may contribute residual confounding. Given the low minor allele frequency (<8%) observed for *TNF-α* and *DAT1* in this cohort, the absence of minor homozygotes remains statistically plausible within the present sample size.

Deviation from Hardy–Weinberg equilibrium (HWE) was observed for *TNF-α* and *DAT1* polymorphisms in this study. This may reflect several factors, including limited sample size, skewed allele frequency distribution, population stratification, or potential genotyping bias associated with HRM analysis. Although genotyping was performed under standardized conditions, subtle genotype misclassification or allelic dropout cannot be entirely excluded. Therefore, these findings should be interpreted with caution. Notably, previous studies have reported that the *TNF-α* (−308) AA genotype may be rare or absent in some populations (Al-Kholy et al., 2016; Al-Rayes et al., 2011), and DAT1 genotype distributions also vary across ethnic groups, including Thai populations (Pattarachotanant et al., 2010; Jones et al., 2019). These observations support that both biological and technical factors may contribute to the observed deviation. Future studies with larger sample sizes and independent validation are warranted.

Overall, these findings support an observational model in which the *TNF-α* (−308G > A) promoter variant is associated with HHV-6 DNA detection in a general Thai community cohort, independent of major measured sociodemographic covariates. Although the observed association between the *TNF-α* polymorphism and HHV-6 DNA detection is biologically plausible, this study does not include functional validation, and therefore causal interpretations should be made with caution. Circulating TNF-α levels are influenced by multiple factors beyond a single genetic variant, including viral load, viral species, co-infections, and host-related factors such as age, sex, comorbidities, and medication use (Turner et al., 2014; Schroder et al., 2004). Consequently, variation in TNF-α levels cannot be attributed solely to a single polymorphism. The findings of this study should therefore be interpreted as reflecting an association with HHV-6 DNA detection rather than a direct mechanistic relationship. Further functional studies are required to validate these observations. Future studies should prioritize longitudinal sampling to distinguish persistent low-level detectability from episodic reactivation, incorporate HHV-6 subtype/strain resolution, and expand immune profiling to clarify how *TNF-α* genetic variation shapes host–virus equilibrium in population settings.

## Data Availability

The datasets presented in this study can be found in online repositories. The names of the repository/repositories and accession number(s) can be found in the article/Supplementary material.

## Supplementary material

SUPPLEMENTARY TABLE S1 Individual-level HHV-6 metagenomic detection, serum TNF-*α* levels, and quantitative HHV-6 viral load among HHV-6–positive participants.

SUPPLEMENTARY TABLE S2 Association between psychological measures (ST-5, Q2, PHQ-9) and genotyped polymorphisms.

SUPPLEMENTARY TABLE S3 Observed and expected genotype counts under Hardy–Weinberg equilibrium (HWE).

SUPPLEMENTARY TABLE S4 Proteomic clinicopathological data of patients.

## References

[ref1] Al-Kholy W. Elsaid A. Sleem A. Fathy H. Elshazli R. Settin A. (2016). TNF-α - 308 G > a and IFN-γ + 874 a > T gene polymorphisms in Egyptian patients with lupus erythematosus. Meta Gene 9, 137–141. doi: 10.1016/j.mgene.2016.06.002. 27331019, 27331019 PMC4909826

[ref2] Al-Rayes H. Al-Swailem R. Albelawi M. Arfin M. Al-Asmari A. Tariq M. (2011). TNF-α and TNF-β gene polymorphism in Saudi rheumatoid arthritis patients. Clin. Med. Insights Arthritis Musculoskelet. Disord. 4, CMAMD.S6941–CMAMD.S6963. doi: 10.4137/CMAMD.S6941, 21792343 PMC3140274

[ref3] Bains N. Abdijadid S. (2024). “Major Depressive Disorder,” in StatPearls [Internet], (Treasure Island, FL: StatPearls Publishing).

[ref4] Bon M. A. M. van Oeveren-Dybicz A. van den Bergh F. A. (2000). Genotyping of HLA-B27 by real-time PCR without hybridization probes. Clin. Chem. 46, 1000–1002. doi: 10.1093/clinchem/46.7.1000, 10894847

[ref5] Bumrungthai S. Buddhisa S. Duangjit S. Passorn S. Sumala S. Prakobkaew N. (2024). Association of HHV-6 reactivation and SLC6A3 (C>T, rs40184), BDNF (C>T, rs6265), and JARID2 (G>a, rs9383046) single nucleotide polymorphisms in depression. Biomed. Rep. 21:181. doi: 10.3892/br.2024.1869, 39420919 PMC11484186

[ref6] Busillo J. M. Cidlowski J. A. (2013). The five Rs of glucocorticoid action during inflammation: ready, reinforce, repress, resolve, and restore. Trends Endocrinol. Metab. 24, 109–119. doi: 10.1016/j.tem.2012.11.005, 23312823 PMC3667973

[ref7] Cain D. W. Cidlowski J. A. (2017). Immune regulation by glucocorticoids. Nat. Rev. Immunol. 17, 233–247. doi: 10.1038/nri.2017.1, 28192415 PMC9761406

[ref8] Chen X. Long F. Cai B. Chen X. Chen G. (2017). A novel relationship for schizophrenia, bipolar and major depressive disorder part 5: a hint from chromosome 5 high density association screen. Am. J. Transl. Res. 9, 2473–2491, 28559998 PMC5446530

[ref9] Choi E. H. Lee H. J. Kim D. H. Eun B. W. . (2005). Virological and clinical features of human herpesvirus-6 infection in Korean children. Pediatr. Infect. Dis. J. 24, 481–486. doi: 10.1097/01.inf.0000165397.48153.3f15933555

[ref10] Chomchoei C. Apidechkul T. Keawdounglek V. Wongfu C. Khunthason S. Kullawong N. . (2020). Prevalence of and factors associated with depression among hill tribe individuals aged 30 years and over in Thailand. Heliyon 6:e04273. doi: 10.1016/j.heliyon.2020.e04273, 32613129 PMC7322052

[ref11] Cohen F. Kemeny M. E. Kearney K. A. Zegans L. S. Neuhaus J. M. Conant M. A. (1999). Persistent stress as a predictor of genital herpes recurrence. Arch. Intern. Med. 159, 2430–2436. doi: 10.1001/archinte.159.20.2430. 10665891, 10665891

[ref12] Collot S. Petit B. Bordessoule D. Alain S. Touati M. Denis F. . (2002). Real-time PCR for quantification of human herpesvirus 6 DNA from lymph nodes and saliva. J. Clin. Microbiol. 40, 2445–2451. doi: 10.1128/JCM.40.7.2445-2451, 12089260 PMC120581

[ref13] Dhabhar F. S. (2014). Effects of stress on immune function: the good, the bad, and the beautiful. Immunol. Res. 58, 193–210. doi: 10.1007/s12026-014-8517-0. 24798553, 24798553

[ref14] Dhabhar F. S. Malarkey W. B. Neri E. Bruce S. (2012). McEwen.Stress-induced redistribution of immune cells-from barracks to boulevards to battlefields: a tale of three hormones. Psychoneuroendocrinology 37, 1345–1368. doi: 10.1016/j.psyneuen.2012.05.008, 22727761 PMC3412918

[ref16] Faris A. Hadi Bin M. Yusof H. Zainal Abidin S. . (2018). Development and validation of high resolution melting assays for high-throughput screening of BDNF rs6265 and DAT1 rs40184. Malay. J. Med. Health Sci. 14, 2636–9346.

[ref17] Ising M. Holsboer F. (2006). Genetics of stress response and stress-related disorders. Dialogues Clin. Neurosci. 8, 433–444. doi: 10.31887/DCNS.2006.8.4/mising. 17290801, 17290801 PMC3181835

[ref18] Jones C. (2023). Intimate relationship between stress and human alpha herpes virus 1 (HSV 1) reactivation from latency. Curr. Clin. Microbiol. Rep. 10, 236–245. doi: 10.1007/s40588-023-00202-9, 38173564 PMC10764003

[ref19] Jones S. E. Tyrrell J. Wood A. R. Beaumont R. N. Ruth K. S. Tuke M. A. . (2019). Genome-wide association analyses of chronotype in 697,828 individuals provides insights into circadian rhythms. Nat Commun. 10:343. doi: 10.1038/s41467-018-08259-7, 30696823 PMC6351539

[ref20] Malhi G. S. Mann J. J. (2018). Depression. Lancet 392, 2299–2312. doi: 10.1016/S0140-6736(18)31948-2, 30396512

[ref21] Noisakran S. Halford W. P. Veress L. Carr D. J. J. (1998). Role of the hypothalmic pituitary adrenal axis and IL-6 in stress-induced reactivation of latent herpes simplex virus type 1. J. Immunol. 160, 5441–5447. doi: 10.4049/jimmunol.160.11.5441, 9605146

[ref22] Oakley R. H. Cidlowski J. A. (2013). The biology of the glucocorticoid receptor: new signaling mechanisms in health and disease. J. Allergy Clin. Immunol. 132, 1033–1044. doi: 10.1016/j.jaci.2013.09.007, 24084075 PMC4084612

[ref23] Padgett D. A. Sherida J. F. Dorne J. Berntson G. G. Candelora J. Glaser R. (1998). Social stress and the reactivation of latent herpes simplex virus type 1. Proc. Natl. Acad. Sci. USA 95, 7231–7235. doi: 10.1073/pnas.95.12.7231, 9618568 PMC22787

[ref24] Paramee R. (2026). The prevalence and risk factors of depression among AIDS patients in mae sai hospital. Available online at: https://he01.tci-thaijo.org/index.php/crmjournal/article/view/240278 (Accessed on January 20, 2026).

[ref25] Pattarachotanant N. Sritharathikhun T. Suttirat S. Tencomnao T. (2010). Association of C/T polymorphism in intron 14 of the dopamine transporter gene (rs40184) with major depression in a northeastern Thai population. Genet. Mol. Res. 9, 835–842. doi: 10.4238/vol9-2gmr767, 20391341

[ref26] Pellett P. E. Ablashi D. V. Ambros P. F. Agut H. Chadburn A. Clark D. A. . (2012). Chromosomally integrated human herpesvirus 6: questions and answers. Rev. Med. Virol. 22, 144–155. doi: 10.1002/rmv.715, 22052666 PMC3498727

[ref27] Pongpakdeesakul S. Ekalaksananan T. Pientong C. Iamchuen N. Buddhisa S. Mahingsa K. . (2023). Human oncogenic Epstein–Barr virus in water and human blood infection of communities in Phayao Province, Thailand. Water 15:323. doi: 10.3390/w15020323

[ref28] Rhen T. Cidlowski J. A. (2005). Antiinflammatory action of glucocorticoids - new mechanisms of old drugs. New England J Med 353, 1711–1723. doi: 10.1056/NEJMra050541, 16236742

[ref29] Schroder K. Hertzog P. J. Ravasi T. Hume D. A. (2004). Interferon-γ: an overview of signals, mechanisms and functions. J. Leukoc. Biol. 75, 163–189. doi: 10.1189/jlb.0603252, 14525967

[ref31] Sumala S. Ekalaksananan T. Pientong C. Buddhisa S. Passorn S. Duangjit S. . (2023). The association of HHV-6 and the TNF-α (−308G/a) promotor with major depressive disorder patients and healthy controls in Thailand. Viruses 15:1898. doi: 10.3390/v15091898. 37766304, 37766304 PMC10535374

[ref32] Tamai S. Hiraoka H. Shimizu K. Miyake K. Hoshi D. Aoki K. . (2022). Variabilities of salivary human herpesvirus 6/7 and cortisol levels during a three-day training camp in judo athletes. J. Physic. Fitness Sports Med. 11, 43–49. doi: 10.7600/jpfsm.11.43

[ref33] Tran X. D. Hoang V. T. Dang T. T. D. Vu T. P. To M. M. Tran T. K. . (2023). Aetiology of acute undifferentiated fever among children under the age of five in Vietnam: a prospective study. J Epidemiol Glob Health 13, 163–172. doi: 10.1007/s44197-023-00121-4, 37258852 PMC10231849

[ref34] Turner M. D. Nedjai B. Hurst T. Pen-On Y. (2014). Cytokines and chemokines: at the crossroads of cell signalling and inflammatory disease. Biochim. Biophys. Acta 1843, 2563–2582. doi: 10.1016/j.bbamcr.2014.05.014, 24892271

[ref35] Vasileios A. Leventaki V. Rassidakis G. Z. Claret F. X. (2019). AP-1 transcription factors as regulators of immune responses in cancer. Cancer 11:1037. doi: 10.3390/cancers11071037, 31340499 PMC6678392

[ref36] Ward K. N. Leong H. N. Nacheva E. P. Howard J. Atkinson C. E. Davies N. W. S. . (2006). Human herpesvirus 6 chromosomal integration in immunocompetent patients results in high levels of viral DNA in blood, sera, and hair follicles. J. Clin. Microbiol. 44, 1571–1574. doi: 10.1128/JCM.44.4.1571-1574, 16597897 PMC1448653

[ref37] Yamanishi K. Shiraki K. Kondo T. Okuno T. . (1990). Seroepidemiological and virological evidence for human herpesvirus-6 infection in infants and children. J. Infect. Dis. 162, 1014–1019. doi: 10.1093/infdis/162.5.1014

[ref38] Yang B. Huang X. Ruan L. Yu T. Li X. Jesse F. F. . (2014). No association of SLC6A3 and SLC6A4 gene polymorphisms with schizophrenia in the Han Chinese population. Neurosci. Lett. 2014579, 114–118. doi: 10.1016/j.neulet.2014.07.004, 25019689

[ref39] Yang L. Zhao Y. Wang Y. Liu L. Zhang X. Li B. . (2015). The effects of psychological stress on depression. Curr. Neuropharmacol. 13, 494–504. doi: 10.2174/1570159x1304150831150507. 26412069, 26412069 PMC4790405

[ref40] Zhang J.-M. An J. (2007). Cytokines, inflammation and pain. Int. Anesthesiol. Clin. 45, 27–37. doi: 10.1097/AIA.0b013e318034194e, 17426506 PMC2785020

[ref41] Zhang E. Ma Y. Yu K. Zheng Y. . (2003). Prevalence of human herpesvirus-6 and -7 infection in healthy Chinese adults: evidence of viral DNA persistence in peripheral blood. J. Med. Virol. 70, 470–474. doi: 10.1002/jmv.10419, 12767013

[ref42] Zheng Y. Zhao Y. Wang Y. Rao J. (2021). A multiplex real-time PCR quantitation of human herpesvirus-6, 7, 8 viruses: application in blood transfusions. Virol. J. 18:38. doi: 10.1186/s12985-021-01510-6. 33602271, 33602271 PMC7891017

