# Supplementary material for: Association between TNF-α (−308G > A) promoter polymorphism and HHV-6 DNA detection in a community-based Thai cohort
Source: Front Microbiol. 2026 Jun 17;17:1825548. doi: 10.3389/fmicb.2026.1825548 (PMC13318981; doi:10.3389/fmicb.2026.1825548)
Supplement: SUPPLEMENTARY TABLE S2 — Association between psychological measures (ST-5, Q2, PHQ-9) and genotyped polymorphisms. [file Table_2.DOCX]

**Supplementary Table S2.** Association between psychological measures (ST-5, Q2, PHQ-9) and genotyped polymorphisms. Genotype distributions of TNF-α (rs1800629), DAT1 (rs40184), JARID2 (rs9383046), and SLC6A3 (rs6345) are presented across categories of stress (ST-5), depression screening (Q2), and depressive symptom severity (PHQ-9). Values are shown as number (percentage) within each psychological category. P-values were calculated using Pearson’s chi-squared test. No statistically significant associations were observed between psychological measures and any of the investigated polymorphisms. Minor genotype counts were low for JARID2 and SLC6A3, and results should therefore be interpreted descriptively.

|  |  |  | *TNF-α* |  |  |  | *DAT1* |  |  |  | *JARID2* |  |  | *SLC6A3* |
| --- | --- | --- | --- | --- | --- | --- | --- | --- | --- | --- | --- | --- | --- | --- |
|  | level | G>A | G | Total |  | C>T | C | Total |  | G>A | G | Total |  | C |
| ST-5 | 0–4 | 97 (14.92) | 553 (85.08) | 650 |  | 153 (24.56) | 470 (75.44) | 623 |  | 2 (0.3) | 669 (99.7) | 671 |  | 593 (100) |
|  | 5–7 | 20 (15.38) | 110 (84.62) | 130 |  | 35 (27.78) | 91 (72.22) | 126 |  | 0 (0) | 134 (100) | 134 |  | 119 (100) |
|  | 8–9 | 2 (11.76) | 15 (88.24) | 17 |  | 3 (17.65) | 14 (82.35) | 17 |  | 0 (0) | 16 (100) | 16 |  | 15 (100) |
|  | 10–15 | 2 (11.76) | 15 (88.24) | 17 |  | 3 (17.65) | 14 (82.35) | 17 |  | 0 (0) | 17 (100) | 17 |  | 16 (100) |
| Total |  | 121 (14.86) | 693 (85.14) | 814 |  | 194 (24.78) | 589 (75.22) | 783 |  | 2 (0.24) | 836 (99.76) | 838 |  | 743 (100) |
| *p*-value |  | 0.962 | | |  | 0.670 | | |  | 0.919 | | |  |  |
| Q2 |  | 24 (13.95) | 148 (86.05) | 172 |  | 45 (26.95) | 122 (73.05) | 167 |  | 0 (0) | 176 (100) | 176 |  | 154 (100) |
|  |  | 95 (15.13) | 533 (84.87) | 628 |  | 143 (23.75) | 459 (76.25) | 602 |  | 2 (0.31) | 645 (99.69) | 647 |  | 576 (100) |
| Total |  | 119 (14.88) | 681 (85.12) | 800 |  | 188 (24.45) | 581 (75.55) | 769 |  | 2 (0.24) | 821 (99.76) | 823 |  | 730 (100) |
| *p*-value |  | 0.701 | | |  | 0.396 | | |  | 0.460 | | |  |  |
| PHQ-9 | 0–6 | 113 (14.95) | 643 (85.05) | 756 |  | 182 (25.07) | 544 (74.93) | 726 |  | 2 (0.26) | 778 (99.74) | 780 |  | 694 (100) |
|  | 7–12 | 8 (16) | 42 (84.0) | 50 |  | 10 (20.41) | 39 (79.59) | 49 |  | 0 (0) | 51 (100) | 51 |  | 43 (100) |
|  | 13–18 | 0 (0) | 10 (100) | 10 |  | 1 (10) | 9 (90) | 10 |  | 0 (0) | 9 (100) | 9 |  | 10 (100) |
|  | 19–30 | 0 (0) | 3 (100) | 3 |  | 1 (33.33) | 2 (66.67) | 3 |  | 0 (0) | 3 (100) | 3 |  | 2 (100) |
| Total |  | 121(14.77) | 698 (85.23) | 819 |  | 194 (24.62) | 594 (75.38) | 788 |  | 2 (0) | 841 (99.29) | 843 |  | 749 (100) |
| *p*-value |  | 0.507 | | |  | 0.610 | | |  | 0.983 | | |  |  |
