# Supplementary material for: Association between TNF-α (−308G > A) promoter polymorphism and HHV-6 DNA detection in a community-based Thai cohort
Source: Front Microbiol. 2026 Jun 17;17:1825548. doi: 10.3389/fmicb.2026.1825548 (PMC13318981; doi:10.3389/fmicb.2026.1825548)
Supplement: SUPPLEMENTARY TABLE S3 — Observed and expected genotype counts under Hardy–Weinberg equilibrium (HWE). [file Table_3.DOCX]

**Supplementary Table S3.** Observed and expected genotype counts under Hardy–Weinberg equilibrium (HWE). Observed and expected genotype frequencies for TNF-α (rs1800629) and DAT1 (rs40184) were calculated using allele frequencies derived from the study cohort. Expected counts were computed under Hardy–Weinberg assumptions (p², 2pq, q²). Deviation from equilibrium was assessed using exact tests as described in the main text. The absence of minor homozygotes contributed to deviation from HWE for TNF-α and DAT1.

| SNP | Genotype | Observed (n) | Expected (n) |
| --- | --- | --- | --- |
| TNF-α (rs1800629) | G/G | 700 | 704.9 |
|  | G/A | 121 | 112.1 |
|  | A/A | 0 | 4.46 |
| DAT1 (rs40184) | C/C | 594 | 605.9 |
|  | C/T | 194 | 169.8 |
|  | T/T | 0 | 11.9 |

Note: Expected counts were calculated using allele frequencies derived from observed genotype data under Hardy–Weinberg assumptions (p², 2pq, q²).
