# Supplementary material for: Association between TNF-α (−308G > A) promoter polymorphism and HHV-6 DNA detection in a community-based Thai cohort
Source: Front Microbiol. 2026 Jun 17;17:1825548. doi: 10.3389/fmicb.2026.1825548 (PMC13318981; doi:10.3389/fmicb.2026.1825548)
Supplement: SUPPLEMENTARY TABLE S4 — Proteomic clinicopathological data of patients. [file Table_4.DOCX]

**Supplement Table S4.** Proteomic clinicopathological data of patients

| Proteomic group | condition | viral load  (copies/µL DNA extract) | TNF level  (pg/mL) | sex | age |
| --- | --- | --- | --- | --- | --- |
| G1 | stress HHV6 TNF pos | #N/A | 0 | 2 | 54 |
| G1 | stress HHV6 TNF pos | 6057426 | 0 | 2 | 58 |
| G1 | stress HHV6 TNF pos | 5212547 | 0 | 1 | 60 |
| G1 | stress HHV6 TNF pos | #N/A | 0 | 2 | 52 |
| G1 | stress HHV6 TNF pos | #N/A | 0 | 2 | 59 |
| G1 | stress HHV6 TNF pos | 7219026 | 0 | 2 | 59 |
| G1 | stress HHV6 TNF pos | 11631117 | 0 | 2 | 61 |
| G1 | stress HHV6 TNF pos | #N/A | 0 | 1 | 61 |
| G2 | stress HHV6 TNF neg | 4064010 | 0 | 2 | 57 |
| G2 | stress HHV6 TNF neg | 16037817 | 1.073495 | 1 | 42 |
| G2 | stress HHV6 TNF neg | 3906717 | 0 | 2 | 38 |
| G2 | stress HHV6 TNF neg | #N/A | 0 | 1 | 58 |
| G2 | stress HHV6 TNF neg | #N/A | 0.521093 | 1 | 46 |
| G2 | stress HHV6 TNF neg | #N/A | 0.645942 | 2 | 52 |
| G2 | stress HHV6 TNF neg | 11144229 | 0 | 2 | 59 |
| G2 | stress HHV6 TNF neg | 5218266 | 0 | 2 | 57 |
| G2 | stress HHV6 TNF neg | 4886015 | 0 | 2 | 59 |
| G2 | stress HHV6 TNF neg | 10119178 | 0 | 2 | 67 |
| G2 | stress HHV6 TNF neg | 3690201 | 0 | 2 | 70 |
| G5 | no stress HHV6 TNF pos | #N/A | 0 | 1 | 59 |
| G5 | no stress HHV6 TNF pos | 1962295 | 0 | 1 | 68 |
| G5 | no stress HHV6 TNF pos | #N/A | 0 | 2 | 53 |
| G5 | no stress HHV6 TNF pos | #N/A | 2.030112 | 2 | 55 |
| G5 | no stress HHV6 TNF pos | 10807287 | 0 | 1 | 58 |
| G5 | no stress HHV6 TNF pos | 0 | 0 | 1 | 70 |
| G5 | no stress HHV6 TNF pos | #N/A | 0.004509 | 2 | 45 |
| G5 | no stress HHV6 TNF pos | #N/A | 0 | 1 | 50 |
| G5 | no stress HHV6 TNF pos | #N/A | 0 | 2 | 52 |
| G5 | no stress HHV6 TNF pos | #N/A | 0 | 2 | 58 |
| G5 | no stress HHV6 TNF pos | #N/A | 0.355295 | 2 | 51 |
| G5 | no stress HHV6 TNF pos | #N/A | 0 | 2 | 52 |
| G5 | no stress HHV6 TNF pos | 14246779 | 0 | 2 | 58 |
| G5 | no stress HHV6 TNF pos | #N/A | 0 | 2 | 58 |
| G5 | no stress HHV6 TNF pos | #N/A | 0 | 2 | 59 |
| G5 | no stress HHV6 TNF pos | 19113302 | 3.245535 | 2 | 57 |
| G5 | no stress HHV6 TNF pos | 6455162 | 0 | 1 | 59 |
| G5 | no stress HHV6 TNF pos | 8198165 | 0 | 2 | 56 |
| G5 | no stress HHV6 TNF pos | #N/A | 0 | 1 | 68 |
| G5 | no stress HHV6 TNF pos | 6620024 | 0 | 2 | 65 |
| G5 | no stress HHV6 TNF pos | 9474882 | 0 | 1 | 66 |
| G5 | no stress HHV6 TNF pos | 4397851 | 1.103679 | 2 | 63 |
| G5 | no stress HHV6 TNF pos | 4264883 | 0.756229 | 1 | 66 |
| G5 | no stress HHV6 TNF pos | 5659321 | 0 | 1 | 63 |
| G5 | no stress HHV6 TNF pos | 3763756 | 0 | 2 | 62 |
| G5 | no stress HHV6 TNF pos | 4554897 | 0 | 2 | 61 |
| G5 | no stress HHV6 TNF pos | #N/A | 0 | 1 | 62 |
| G5 | no stress HHV6 TNF pos | 4505227 | 61.86552 | 1 | 72 |
| G6 | no stress HHV6 TNF neg | #N/A | 4.159728 | 2 | 52 |
| G6 | no stress HHV6 TNF neg | #N/A | 0.644057 | 2 | 53 |
| G6 | no stress HHV6 TNF neg | 2861369 | 0 | 1 | 69 |
| G6 | no stress HHV6 TNF neg | 5548721 | 0 | 2 | 36 |
| G6 | no stress HHV6 TNF neg | #N/A | 0 | 2 | 48 |
| G6 | no stress HHV6 TNF neg | 5721715 | 0 | 2 | 55 |
| G6 | no stress HHV6 TNF neg | #N/A | 0.478019 | 1 | 56 |
| G6 | no stress HHV6 TNF neg | #N/A | 0.631504 | 2 | 55 |
| G6 | no stress HHV6 TNF neg | #N/A | 0 | 2 | 59 |
| G6 | no stress HHV6 TNF neg | 5172693 | 0 | 2 | 46 |
| G6 | no stress HHV6 TNF neg | #N/A | 0 | 2 | 53 |
| G6 | no stress HHV6 TNF neg | 0 | 0 | 2 | 53 |
| G6 | no stress HHV6 TNF neg | 9392134 | 0 | 2 | 57 |
| G6 | no stress HHV6 TNF neg | 6329009 | 0 | 2 | 55 |
| G6 | no stress HHV6 TNF neg | 3137428 | 4.866426 | 2 | 67 |
| G6 | no stress HHV6 TNF neg | 0 | 0.891219 | 2 | 36 |
| G6 | no stress HHV6 TNF neg | #N/A | 0 | 2 | 44 |
| G6 | no stress HHV6 TNF neg | #N/A | 0 | 2 | 42 |
| G6 | no stress HHV6 TNF neg | #N/A | 0 | 1 | 48 |
| G6 | no stress HHV6 TNF neg | #N/A | 0.883392 | 1 | 49 |
| G6 | no stress HHV6 TNF neg | 6110794 | 0 | 2 | 41 |
| G6 | no stress HHV6 TNF neg | #N/A | 0.853409 | 2 | 53 |
| G6 | no stress HHV6 TNF neg | #N/A | 0 | 1 | 55 |
| G6 | no stress HHV6 TNF neg | #N/A | 0 | 1 | 54 |
| G6 | no stress HHV6 TNF neg | #N/A | 0 | 2 | 60 |
| G6 | no stress HHV6 TNF neg | #N/A | 0 | 2 | 59 |
| G6 | no stress HHV6 TNF neg | #N/A | 0.506895 | 1 | 59 |
| G6 | no stress HHV6 TNF neg | 0 | 1.019447 | 1 | 56 |
| G3 | stress HHV6 neg TNF pos | #N/A | 0 | 2 | 65 |
| G3 | stress HHV6 neg TNF pos | #N/A | 0 | 2 | 50 |
| G3 | stress HHV6 neg TNF pos | #N/A | 0 | 2 | 54 |
| G3 | stress HHV6 neg TNF pos | #N/A | 4.408132 | 2 | 29 |
| G3 | stress HHV6 neg TNF pos | #N/A | 0 | 2 | 26 |
| G3 | stress HHV6 neg TNF pos | #N/A | 1.107502 | 1 | 66 |
| G3 | stress HHV6 neg TNF pos | #N/A | 1.336286 | 2 | 22 |
| G3 | stress HHV6 neg TNF pos | #N/A | 0 | 2 | 27 |
| G3 | stress HHV6 neg TNF pos | #N/A | 2.87146 | 2 | 42 |
| G3 | stress HHV6 neg TNF pos | #N/A | 0 | 1 | 67 |
| G3 | stress HHV6 neg TNF pos | #N/A | 0 | 1 | 63 |
| G3 | stress HHV6 neg TNF pos | #N/A | 0.39379 | 2 | 64 |
| G3 | stress HHV6 neg TNF pos | #N/A | 0 | 2 | 71 |
| G3 | stress HHV6 neg TNF pos | #N/A | 90.07128 | 2 | 67 |
| G3 | stress HHV6 neg TNF pos | #N/A | 0 | 2 | 23 |
| G3 | stress HHV6 neg TNF pos | #N/A | 0.783324 | 2 | 61 |
| G4 | stress HHV6 neg TNF neg | #N/A | 0.713104 | 1 | 9 |
| G4 | stress HHV6 neg TNF neg | #N/A | 0 | 2 | 19 |
| G4 | stress HHV6 neg TNF neg | #N/A | 0 | 2 | 22 |
| G4 | stress HHV6 neg TNF neg | #N/A | 0 | 2 | 30 |
| G4 | stress HHV6 neg TNF neg | #N/A | 0.458477 | 2 | 31 |
| G4 | stress HHV6 neg TNF neg | #N/A | 0 | 2 | 15 |
| G4 | stress HHV6 neg TNF neg | #N/A | 1.039532 | 2 | 16 |
| G4 | stress HHV6 neg TNF neg | #N/A | 0 | 2 | 40 |
| G4 | stress HHV6 neg TNF neg | #N/A | 2.115711 | 2 | 24 |
| G4 | stress HHV6 neg TNF neg | #N/A | 0.683428 | 2 | 26 |
| G4 | stress HHV6 neg TNF neg | #N/A | 0 | 1 | 21 |
| G4 | stress HHV6 neg TNF neg | #N/A | 0 | 2 | 36 |
| G4 | stress HHV6 neg TNF neg | #N/A | 0 | 1 | 33 |
| G4 | stress HHV6 neg TNF neg | #N/A | 2.775281 | 1 | 40 |
| G4 | stress HHV6 neg TNF neg | #N/A | 0 | 2 | 44 |
| G4 | stress HHV6 neg TNF neg | #N/A | 2.850176 | 2 | 8 |
| G4 | stress HHV6 neg TNF neg | #N/A | 0 | 1 | 16 |
| G4 | stress HHV6 neg TNF neg | #N/A | 19.80964 | 1 | 21 |
| G4 | stress HHV6 neg TNF neg | #N/A | 1.791328 | 1 | 36 |
| G4 | stress HHV6 neg TNF neg | #N/A | 0 | 1 | 39 |
| G4 | stress HHV6 neg TNF neg | #N/A | 3.536759 | 2 | 49 |
| G4 | stress HHV6 neg TNF neg | #N/A | 2.088756 | 2 | 42 |
| G4 | stress HHV6 neg TNF neg | #N/A | 0 | 2 | 60 |
| G4 | stress HHV6 neg TNF neg | #N/A | 2.060776 | 2 | 53 |
| G4 | stress HHV6 neg TNF neg | #N/A | 0 | 2 | 69 |
| G4 | stress HHV6 neg TNF neg | #N/A | 0 | 1 | 71 |
| G4 | stress HHV6 neg TNF neg | #N/A | 0 | 2 | 79 |
| G4 | stress HHV6 neg TNF neg | #N/A | 2.642449 | 2 | 14 |
| G4 | stress HHV6 neg TNF neg | #N/A | 0 | 2 | 21 |
| G7 | no stress HHV6 neg TNF pos | #N/A | 0 | 2 | 50 |
| G7 | no stress HHV6 neg TNF pos | #N/A | 66.09907 | 2 | 56 |
| G7 | no stress HHV6 neg TNF pos | #N/A | 2.687044 | 1 | 57 |
| G7 | no stress HHV6 neg TNF pos | #N/A | 0 | 1 | 64 |
| G7 | no stress HHV6 neg TNF pos | #N/A | 0 | 2 | 44 |
| G7 | no stress HHV6 neg TNF pos | #N/A | 11.943 | 2 | 47 |
| G7 | no stress HHV6 neg TNF pos | #N/A | 0 | 2 | 48 |
| G7 | no stress HHV6 neg TNF pos | #N/A | 2.701343 | 2 | 58 |
| G7 | no stress HHV6 neg TNF pos | #N/A | 5.445316 | 2 | 62 |
| G7 | no stress HHV6 neg TNF pos | #N/A | 0 | 2 | 70 |
| G7 | no stress HHV6 neg TNF pos | #N/A | 0 | 1 | 67 |
| G7 | no stress HHV6 neg TNF pos | #N/A | 0 | 1 | 61 |
| G7 | no stress HHV6 neg TNF pos | #N/A | 0 | 2 | 62 |
| G7 | no stress HHV6 neg TNF pos | #N/A | 0 | 2 | 64 |
| G7 | no stress HHV6 neg TNF pos | #N/A | 0 | 1 | 61 |
| G7 | no stress HHV6 neg TNF pos | #N/A | 0 | 2 | 67 |
| G7 | no stress HHV6 neg TNF pos | #N/A | 1.330091 | 2 | 72 |
| G7 | no stress HHV6 neg TNF pos | #N/A | 3.043066 | 2 | 22 |
| G7 | no stress HHV6 neg TNF pos | #N/A | 0.442823 | 2 | 44 |
| G7 | no stress HHV6 neg TNF pos | #N/A | 7.181985 | 2 | 64 |
| G7 | no stress HHV6 neg TNF pos | #N/A | 0.590112 | 2 | 62 |
| G7 | no stress HHV6 neg TNF pos | #N/A | 0 | 2 | 68 |
| G7 | no stress HHV6 neg TNF pos | #N/A | 0 | 1 | 64 |
| G7 | no stress HHV6 neg TNF pos | #N/A | 0.680721 | 1 | 66 |
| G7 | no stress HHV6 neg TNF pos | #N/A | 0 | 1 | 24 |
| G8 | no stress HHV6 neg TNF neg | #N/A | 0 | 2 | 18 |
| G8 | no stress HHV6 neg TNF neg | #N/A | 0 | 1 | 21 |
| G8 | no stress HHV6 neg TNF neg | #N/A | 0 | 2 | 40 |
| G8 | no stress HHV6 neg TNF neg | #N/A | 1.425312 | 1 | 7 |
| G8 | no stress HHV6 neg TNF neg | #N/A | 0.713104 | 2 | 8 |
| G8 | no stress HHV6 neg TNF neg | #N/A | 0 | 1 | 9 |
| G8 | no stress HHV6 neg TNF neg | #N/A | 0.787292 | 1 | 6 |
| G8 | no stress HHV6 neg TNF neg | #N/A | 7.909823 | 1 | 13 |
| G8 | no stress HHV6 neg TNF neg | #N/A | 0.357 | 2 | 16 |
| G8 | no stress HHV6 neg TNF neg | #N/A | 2.056361 | 2 | 12 |
| G8 | no stress HHV6 neg TNF neg | #N/A | 1.008624 | 2 | 46 |
| G8 | no stress HHV6 neg TNF neg | #N/A | 0 | 1 | 41 |
| G8 | no stress HHV6 neg TNF neg | #N/A | 0 | 2 | 57 |
| G8 | no stress HHV6 neg TNF neg | #N/A | 0 | 2 | 58 |
| G8 | no stress HHV6 neg TNF neg | #N/A | 0 | 2 | 61 |
| G8 | no stress HHV6 neg TNF neg | #N/A | 0 | 2 | 65 |
| G8 | no stress HHV6 neg TNF neg | #N/A | 0 | 2 | 61 |
| G8 | no stress HHV6 neg TNF neg | #N/A | 0 | 2 | 61 |
| G8 | no stress HHV6 neg TNF neg | #N/A | 0 | 2 | 79 |
| G8 | no stress HHV6 neg TNF neg | #N/A | 0 | 1 | 14 |
| G8 | no stress HHV6 neg TNF neg | #N/A | 0 | 2 | 22 |
| G8 | no stress HHV6 neg TNF neg | #N/A | 0.357 | 2 | 21 |
| G8 | no stress HHV6 neg TNF neg | #N/A | 0 | 2 | 21 |
| G8 | no stress HHV6 neg TNF neg | #N/A | 0 | 2 | 38 |
| G8 | no stress HHV6 neg TNF neg | #N/A | 39.87771 | 2 | 42 |
| G8 | no stress HHV6 neg TNF neg | #N/A | 0 | 2 | 47 |
| G8 | no stress HHV6 neg TNF neg | #N/A | 0 | 2 | 57 |

Note: #N/A represented with below detection limit of HHV-6 viral load and this samples were not included in mean viral load analysis. Sex: 1 represented male, 2 represented female.
