# Supplementary material for: Association between TNF-α (−308G > A) promoter polymorphism and HHV-6 DNA detection in a community-based Thai cohort
Source: Front Microbiol. 2026 Jun 17;17:1825548. doi: 10.3389/fmicb.2026.1825548 (PMC13318981; doi:10.3389/fmicb.2026.1825548)
Supplement: Supplementary file 5 [file Supplementary_file_1.DOCX]

**
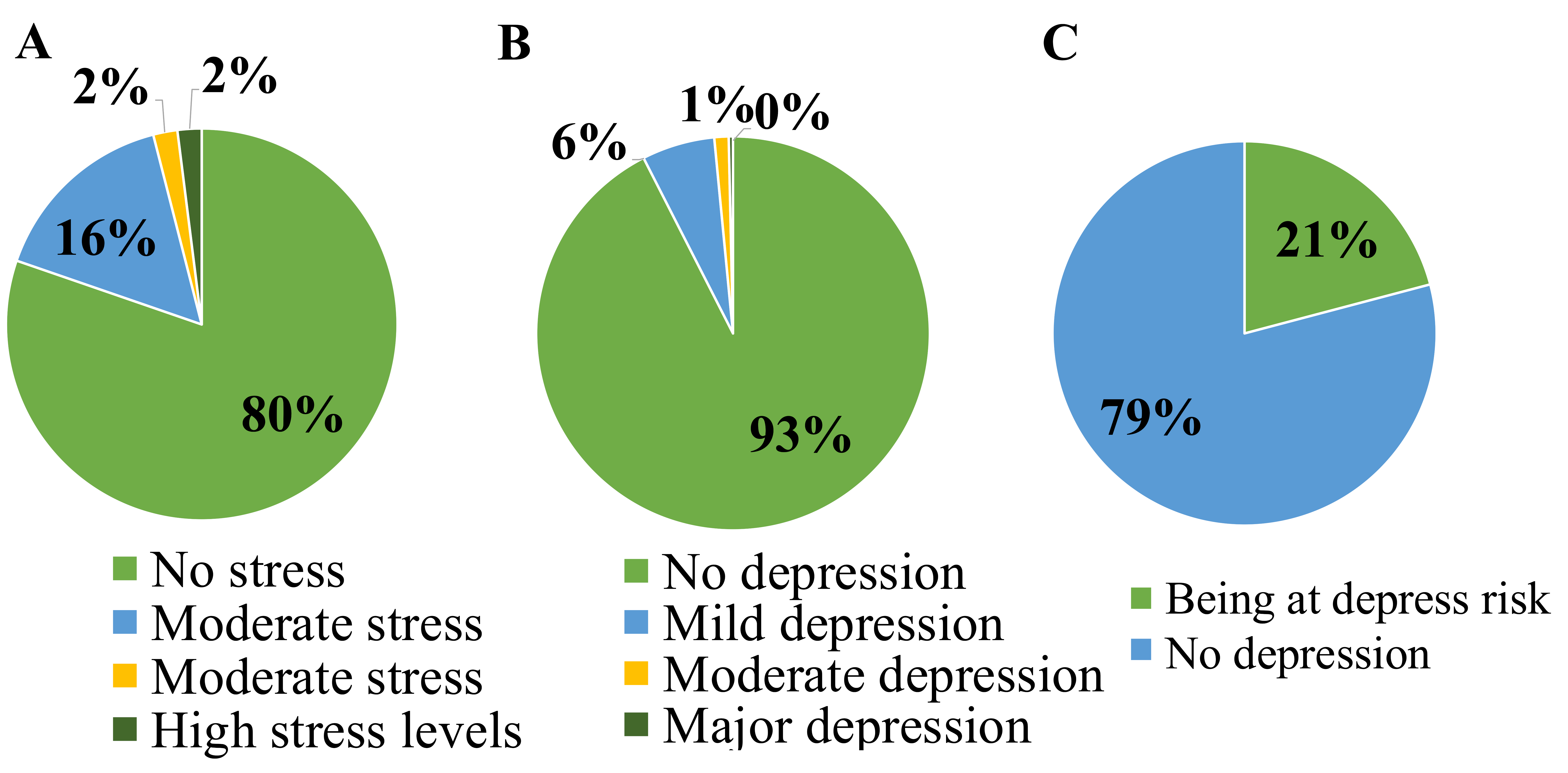
**

**Supplementary Figure S1.** Distribution of psychological stress and depressive symptom scores in the study population (n = 852). (A) Stress levels assessed using the Srithanya Stress Test (ST-5), categorized as no stress (score 0–4), mild-to-moderate stress (5–7), moderate stress (8–9), and high stress (10–15). (B) Depression severity assessed using the Patient Health Questionnaire-9 (PHQ-9), classified as none (0–6), mild (7–12), moderate (13–18), and severe (19–30). (C) Risk of depression assessed using the Depression Questionnaire-2 (Q2), categorized as no risk and at risk of depression according to standard screening cutoffs. Percentages represent the proportion of individuals within the total study population.
